# The Power of Inclusion: Advancing Minority Aging Research in Cognitive Health and Alzheimer’s Disease

**DOI:** 10.1093/geroni/igaf122.1526

**Published:** 2025-12-31

**Authors:** Lisa Barnes

**Affiliations:** Rush University Medical Center, Chicago, Illinois, United States

## Abstract

Lisa L. Barnes, PhD is the Alla V. and Solomon Jesmer Professor of Gerontology and Geriatric Medicine and a cognitive neuropsychologist within the Rush Alzheimer’s Disease Center at Rush University Medical Center. She is also the Associate Director of the Rush Alzheimer’s Disease Research Center. She received her PhD from the University of Michigan in biopsychology and completed a post-doctoral fellowship in cognitive neuroscience at the University of California, Davis. She joined the faculty of Rush as an assistant professor in 1999. Dr. Barnes has received many NIH grants and has published over 300 manuscripts. She is internationally recognized for her contributions to minority aging and minority health. Her research interests include disparities in chronic diseases of aging, cognitive decline, and risk factors for Alzheimer’s disease. She is the Principal Investigator of two longitudinal community-based studies of older African Americans, including the Minority Aging Research Study (MARS), which has been funded by NIA since 2004 and the African American Clinical Core which she has led since 2008. She advocates for recruitment of under-represented groups into clinical studies and has received many awards and fellowships from universities and organizations throughout the U.S.

